# Growth on Chitin Impacts the Transcriptome and Metabolite Profiles of Antibiotic-Producing *Vibrio coralliilyticus* S2052 and *Photobacterium galatheae* S2753

**DOI:** 10.1128/mSystems.00141-16

**Published:** 2017-01-03

**Authors:** Sonia Giubergia, Christopher Phippen, Kristian Fog Nielsen, Lone Gram

**Affiliations:** a NovoNordisk Foundation Centre for Biosustainability, Technical University of Denmark, Kgs. Lyngby, Denmark; b Department of Biotechnology and Biomedicine, Technical University of Denmark, Kgs. Lyngby, Denmark; University of California, San Diego

**Keywords:** *Vibrionaceae*, chitin, regulation, secondary metabolism

## Abstract

Members of the *Vibrionaceae* family are often associated with chitin-containing organisms, and they are thought to play a major role in chitin degradation. The purpose of the present study was to determine how chitin affects the transcriptome and metabolome of two bioactive *Vibrionaceae* strains, *Vibrio coralliilyticus* and *Photobacterium galatheae*. We focused on chitin degradation genes and secondary metabolites based on the assumption that these molecules in nature confer an advantage to the producer. Growth on chitin caused upregulation of genes related to chitin metabolism and of genes potentially involved in host colonization and/or infection. The expression of genes involved in secondary metabolism was also significantly affected by growth on chitin, in one case being 34-fold upregulated. This was reflected in the metabolome, where the antibiotics andrimid and holomycin were produced in larger amounts on chitin. Other polyketide synthase/ nonribosomal peptide synthetase (PKS-NRPS) clusters in *P. galatheae* were also strongly upregulated on chitin. Collectively, this suggests that both *V. coralliilyticus* and *P. galatheae* have a specific lifestyle for growth on chitin and that their secondary metabolites likely play a crucial role during chitin colonization.

**IMPORTANCE** The bacterial family *Vibrionaceae* (vibrios) is considered a major player in the degradation of chitin, the most abundant polymer in the marine environment; however, the majority of studies on the topic have focused on a small number of *Vibrio* species. In this study, we analyzed the genomes of two vibrios to assess their genetic potential for the degradation of chitin. We then used transcriptomics and metabolomics to demonstrate that chitin strongly affects these vibrios at both the transcriptional and metabolic levels. We observed a strong increase in production of secondary metabolites, suggesting an ecological role for these molecules during chitin colonization in the marine environment.

## Abstract (precis)

The bacterial family *Vibrionaceae* (vibrios) is considered a major player in the degradation of chitin, the most abundant polymer in the marine environment; however, the majority of studies on the topic have focused on a small number of *Vibrio* species. In this study, we analyzed the genomes of two vibrios to assess their genetic potential for the degradation of chitin. We then used transcriptomics and metabolomics to demonstrate that chitin strongly affects these vibrios at both the transcriptional and metabolic levels. We observed a strong increase in production of secondary metabolites, suggesting an ecological role for these molecules during chitin colonization in the marine environment.

## INTRODUCTION

Chitin, a polysaccharide composed of *N*-acetylglucosamine (GlcNAc) units, is the most abundant polymer in the marine environment, where it is the primary component of the exoskeleton of zooplankton (1). Members of the *Vibrionaceae* family (vibrios) are often associated with chitinous surfaces (2), and although the ability to metabolize this molecule has been suggested to be an ancestral feature of the whole family (3), characterization of the chitin catabolic pathway has been performed only on a limited number of species from the *Vibrio* genus, mostly *V. cholerae* and *V. furnissii*.

**FIG 1:**
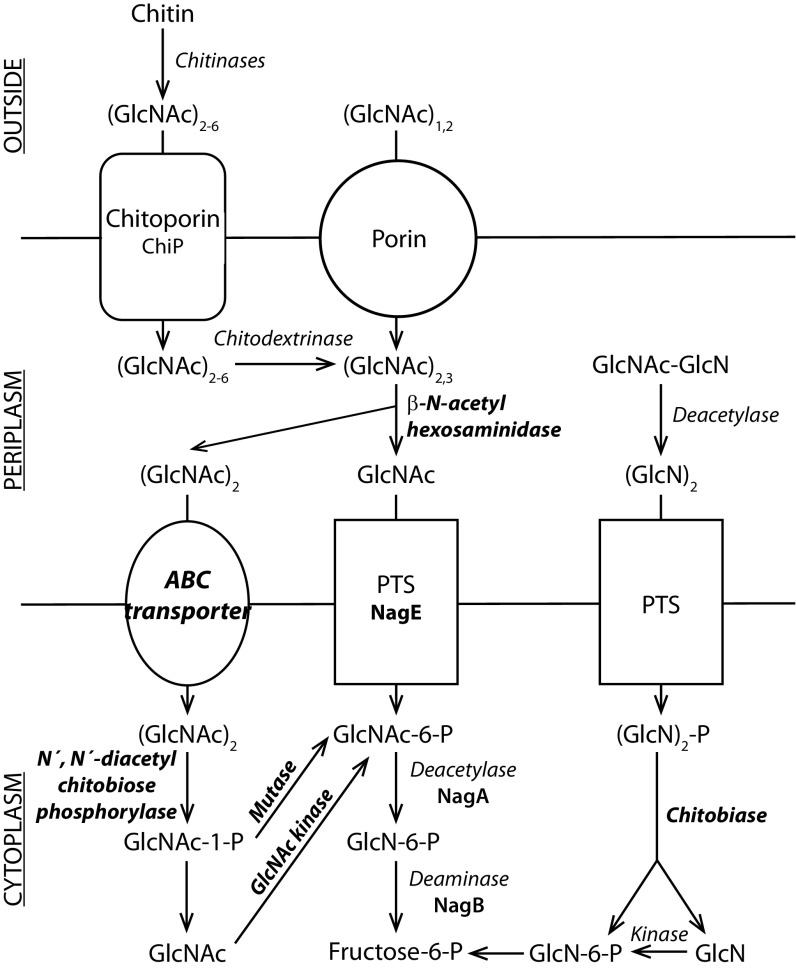
Model for chitin metabolism in vibrios. The enzymes involved in the process are indicated next to reaction arrows. Enzymes and cellular components that are encoded by genes from the (GlcNAc)_2_ operon are labeled in bold italics, while those that are encoded by genes from the *nag* operon are indicated in bold. (Adapted from reference 3 with permission.)

The first steps in the establishment of the bacterium-chitin association rely on a gradient of chitin-derived oligosaccharides released by chitin-containing organisms, which drives bacteria to the chitin surface by chemotaxis (4). This is followed by adhesion of the bacteria to the surface (4). In the chitin utilization model for *V. cholerae* proposed by Hunt and colleagues (3) (Fig. 1), the next step is the secretion of chitinases, enzymes that hydrolyze chitin into GlcNAc oligosaccharides. These oligosaccharides are then transferred into the periplasmic space, where they are further cleaved and/or modified before being transported into the cytoplasm and converted to fructose-6-phosphate, which enters the central metabolism (3). Most of the genes required for the steps of this model occurring in the periplasm and the cytoplasm are organized in the *nag* and (GlcNAc)_2_ operons (Fig. 1). The former has been well characterized in *Escherichia coli* and is controlled by the transcriptional regulator NagC, which represses the operon when no GlcNAc is present in the environment (5, 6). The latter has been identified in *V. cholerae*, and its expression depends on ChiS, a hybrid sensor kinase that is active only when (GlcNAc)_2_ is available (7, 8).

Genome mining has revealed that vibrios harbor the genetic potential for the production of numerous secondary metabolites (9), and several bioactive molecules have been isolated from members of the *Vibrionaceae* family (10). Microbial secondary metabolites are thought to play a role in several ecological phenomena in nature, including antagonism and intercellular communication (11, 12). The coral pathogen *Vibrio coralliilyticus* doubles the production of the antibiotic andrimid per cell during growth on chitin; we hypothesized that the increased production may confer an advantage over competitors during chitin colonization (13).

In this study, we used a multi-omics approach to investigate the influence of chitin on the metabolism of two *Vibrionaceae* strains belonging to different genera of the family, known to produce bioactive metabolites. Analysis of the genomes of *V. coralliilyticus* S2052 and *Photobacterium galatheae* S2753 revealed potential for both the chitin utilization and the biosynthesis of several secondary metabolites. The transcriptomic and metabolite profiles of the two strains grown on chitin revealed insights about cellular components, processes, and small molecules potentially involved in the colonization and degradation of chitinous surfaces in nature.

## RESULTS

### The genetic potential of *V. coralliilyticus* S2052 and *P. galatheae* S2753 for chitin degradation.

**FIG 2:**
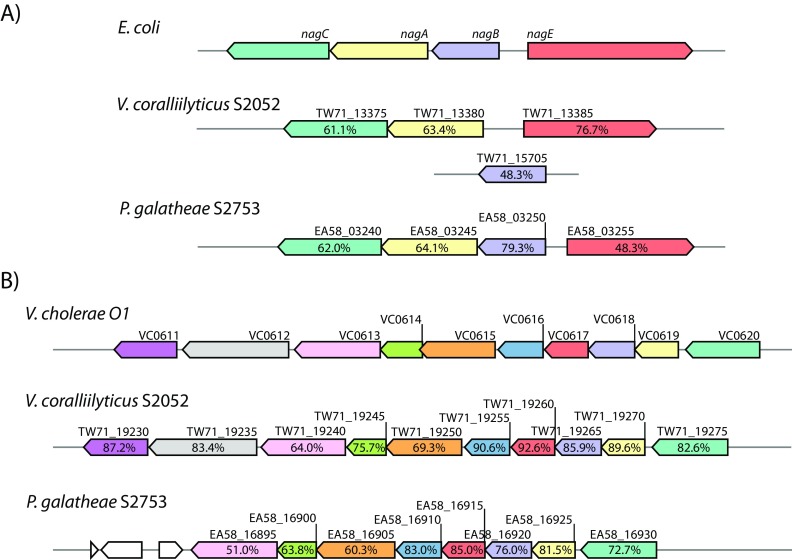
(A) *nag* operons in *E. coli*, from which the operon was originally characterized, *Vibrio coralliilyticus* S2052, and *Photobacterium galatheae* S2753. (B) (GlcNAc)_2_ operons in *V. cholerae* O1, in which the operon was identified first, *V. coralliilyticus* S2052, and *P. galatheae* S2753. The analysis was done using MultiGeneBlast (44). The percentages indicate the percent identity of the translated nucleotide sequences.

We identified 15 and 7 genes in *V. coralliilyticus* S2052 and *P. galatheae* S2753, respectively, whose translated sequences contain one or more Pfam domains involved in the binding of chitin and/or cellulose (Pfam domains CBM_5_12, CBM_12_2, CHB_HEX, chiA_N term, and chiC) and in the hydrolysis of chitin, chitin-derived oligosaccharides, or cellulose (Pfam domains GH3, GH18, GH19, GH20, and LPMO_10) (see Table S1 in the supplemental material). Based on the presence of signal peptides in their amino acid sequences, most of these proteins are likely to be secreted into the extracellular environment, but putative outer membrane and periplasmic proteins were also predicted. In both genomes, one (*P. galatheae* S2753) or more (*V. coralliilyticus* S2052) putative cytoplasmic β-*N*-acetylhexosaminidases, which catalyze the hydrolysis of GlcNAc units from the nonreducing end of chitin, were found. Both genomes contain genes highly homologous to *chiP*, which in *V. furnissii* encodes ChiP, a chitoporin required for the uptake of chitin-derived oligosaccharides (14) (Fig. 1). They also harbor both the *nag* and (GlcNAc)_2_ operons. The organizations of the *nag* operons, however, are different in the two strains. In *P. galatheae* S2753, the four genes included in the operon are adjacent in the genome, whereas in *V. coralliilyticus* S2052, *nagB* is separated from the other genes (Fig. 2). As for the (GlcNAc)_2_ operon (VC0611 to VC0620 in *V. cholerae*), it is completely present in the genome of *V. coralliilyticus* S2052, but we did not detect any gene homolog of VC0611 or VC0612 in the genome of *P. galatheae* S2753 (Fig. 2).

10.1128/mSystems.00141-16.1 TABLE S1  List of genes identified in the genomes of *Vibrio coralliilyticus* S2052 and *Photobacterium galatheae* S2753 and encoded proteins containing Pfam domains related to the binding or the hydrolysis of chitin and cellulose. The predicted cellular localization is indicated as well. DUF, domain of unknown function. Download TABLE S1, DOCX file, 0.04 MB. Copyright © 2017 Giubergia et al. 2017 Giubergia et al. This content is distributed under the terms of the Creative Commons Attribution 4.0 International license.

### *V. coralliilyticus* S2052 and *P. galatheae* S2753 harbor genetic potential for the biosynthesis of secondary metabolites.

Analysis with antiSMASH (15) of the genomes of *V. coralliilyticus* S2052 and *P. galatheae* S2753 found 7 and 13 putative biosynthetic gene clusters (BGCs) for the production of secondary metabolites, respectively (Table S2). Several of the predicted gene clusters included polyketide synthases (PKSs) and/or nonribosomal peptide synthetases (NRPSs), which were present in 5 of the BGCs from *V. coralliilyticus* S2052 and in 7 of the BGCs from *P. galatheae* S2753. Both genomes harbor a putative siderophore BGC and have the genetic potential for the production of the osmolyte ectoine. One putative gene cluster for bacteriocin production was predicted by antiSMASH in both genomes; however, BAGEL3 and Pfam domain analyses did not confirm these results (not shown).

10.1128/mSystems.00141-16.2 TABLE S2  antiSMASH prediction of the genetic potential of *Vibrio coralliilyticus* S2052 and *Photobacterium galatheae* S2753 for the biosynthesis of secondary metabolites. The table lists the types of putative biosynthetic gene cluster (BGCs), the locus tags of the genes predicted to be part of them, and, when known, the most similar known biosynthetic gene cluster. T3PKS, type 3 polyketide synthase; NRPS, nonribosomal peptide synthase; T1PKS, type 1 polyketide synthase. Download Table S2, DOCX file, 0.04 MB. Copyright © 2017 Giubergia et al. 2017 Giubergia et al. This content is distributed under the terms of the Creative Commons Attribution 4.0 International license.

### Global transcription profile of *V. coralliilyticus* S2052 grown on chitin.

**FIG 3:**
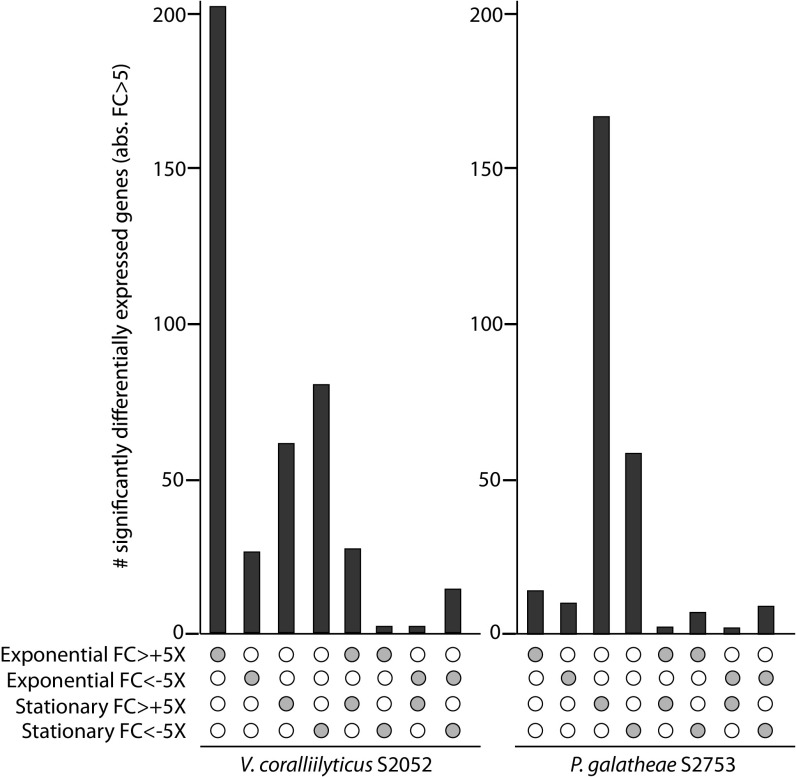
Matrix layout of the differentially expressed genes (≥5-fold change, *P* value < 0.05, *q* value < 0.05) for all intersections. A single gray circle in the lower part of the figure indicates that the number of genes represented by the corresponding bar was differentially expressed only under one condition, while two gray circles indicate that genes were differentially expressed under two conditions. FC, fold change; abs., absolute.

We mapped 97.7% (±1.5%) and 83.40% (±3.5%) of the sequencing reads to the reference genomes of *V. coralliilyticus* S2052 and *P. galatheae* S2753, respectively. After statistical analysis, we evaluated the up- and downregulation (absolute fold change, >5) of the genes when the strains were grown on chitin compared to when they were grown on glucose (Fig. 3). In *V. coralliilyticus* S2052, 231 genes were significantly upregulated and 42 were downregulated when cultures were harvested in the exponential phase, whereas 90 genes were upregulated and 96 were downregulated when RNA samples were prepared from cultures in the stationary phase. Genes for proteins that are part of the respiratory chain and for components of the type III secretion system were downregulated at both samplings. Genes related to host colonization (e.g., genes encoding proteins containing the hemolysin-coregulated protein [HCP] effector domain or that are involved in the production of R-bodies) were upregulated both in the exponential and the stationary phase. The same observation was made for genes involved in natural competence (e.g., TW71_22895). Genes that were upregulated only in the exponential phase included genes encoding components of transporters, including C_4_-dicarboxylate ABC transporters, for enzymes involved in fatty acid degradation and for proteins required for the synthesis of the polyhydroxyalkanoate (PHA) storage compounds. Genes related to adhesion and biofilm formation, including pilus assembly and production of cell capsule polysaccharides and exopolysaccharides, were upregulated on chitin in the stationary phase.

### Global transcription profile of *P. galatheae* S2753 grown on chitin.

For *P. galatheae* S2753, 23 genes were significantly upregulated and 21 were downregulated in the exponential phase, while 171 and 74 genes were up- and downregulated, respectively, when cultures reached stationary phase (Fig. 3). Many of the genes (42%) predicted in the genome of this strain were annotated as “hypothetical protein,” and unfortunately, the use of alternative annotations and additional information as described in Materials and Methods did not facilitate more useful analysis. However, genes related to the respiratory chain were downregulated and genes involved in histidine metabolism were upregulated in both exponential and stationary phase. Genes related to fatty acid degradation, phosphate uptake, and the biosynthesis of aromatic amino acids were upregulated in stationary phase when the strain was grown on chitin, as were genes encoding proteins containing Pfam domains related to the biosynthesis of lipoproteins. Although in *V. coralliilyticus* S2052, the most upregulated genes in the stationary phase were related to chitin utilization, in *P. galatheae* S2753, the most significantly differentially expressed genes were the EA_20780 (encoding a putative benzoylformate decarboxylase) and EA_20785 (encoding a hypothetical protein including a flavin-containing amine oxidoreductase Pfam domain) genes (Table S3).

10.1128/mSystems.00141-16.3 TABLE S3  The 10 most up- and downregulated genes for each strain under each condition at the two sampling points (late exponential and stationary phase). Values refer to growth on chitin compared to on glucose. Up, upregulated; down, downregulated; FC, fold change. Download TABLE S3, DOCX file, 0.03 MB. Copyright © 2017 Giubergia et al. 2017 Giubergia et al. This content is distributed under the terms of the Creative Commons Attribution 4.0 International license.

### Chitin utilization-related genes are significantly upregulated in exponential phase and stationary phase when *V. coralliilyticus* S2052 is grown on chitin.

**FIG 4:**
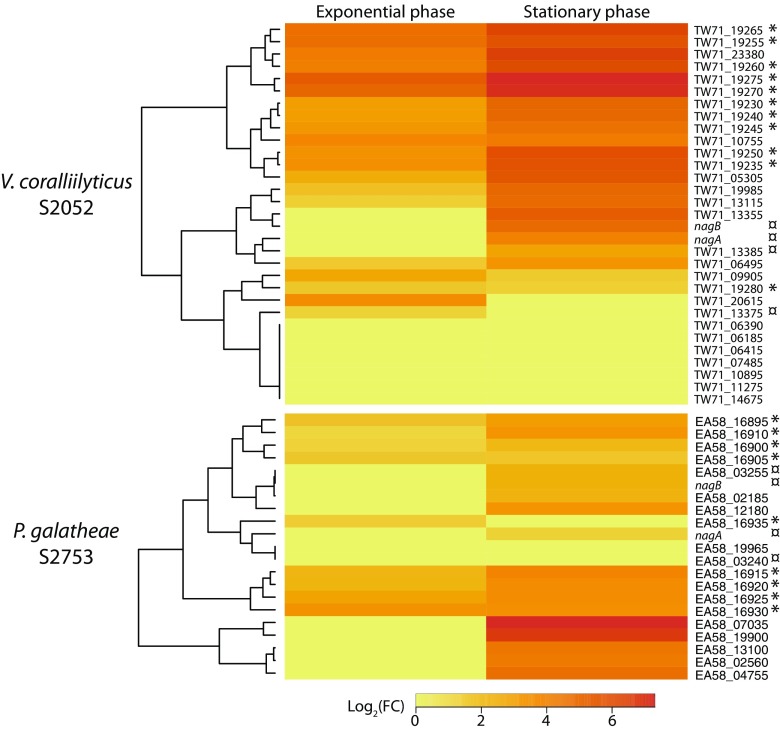
Heat map and hierarchical cluster analysis of the fold changes of genes related to chitin utilization identified in the genome of *Vibrio coralliilyticus* S2052 (top) and *Photobacterium galatheae* S2753 (bottom) at the two time points analyzed in this study (exponential and stationary phases). FC, fold change; *, genes from the (GlcNAc)2 operon plus *chiS* homolog; ¤, genes from the *nag* operon.

In *V. coralliilyticus* S2052, 8 of the 14 genes that do not belong to the (GlcNAc)_2_ or to the *nag* operon but that we predicted to be required for chitin utilization were upregulated on chitin during exponential phase and stationary phase (Fig. 4). The TW71_20615 gene, encoding a putative porin, was upregulated in the exponential but not in the stationary phase. In contrast, the TW71_13355 gene, encoding a putative β-*N*-acetylhexosaminidase, was significantly upregulated on chitin only in the stationary phase. The (GlcNAc)_2_ operon was upregulated both in the late exponential and in the stationary phase. With respect to the *nag* operon, the homolog of the NagC transcriptional regulator-encoding gene (TW71_13375) was positively differentially expressed at both time points, while the rest of the operon was upregulated only in the stationary phase.

### Chitin utilization-related genes are upregulated mostly in the late stages of growth on chitin in *P. galatheae* 2753.

All the genes predicted to be related to chitin utilization besides the (GlcNAc)_2_ and *nag* operons were positively differentially expressed in the stationary phase except EA58_19965, which was not differentially expressed at any time point. The (GlcNAc)_2_ operon was upregulated both in exponential and stationary phase (Fig. 4). As for the *nag* operon in *P. galatheae* S2753, *nagA*, *nagB*, and the homolog of *nagE* were upregulated only in the stationary phase, while the gene encoding the transcriptional regulator (EA58_03240) was not significantly differentially expressed in the exponential or in the stationary phase.

### The BGCs of *V. coralliilyticus* S2052 undergo both up- and downregulation on chitin compared to on glucose.

When we looked at the fold changes in the expression levels of the putative biosynthetic genes that are part of the BGCs predicted by antiSMASH in the genomes of *V. coralliilyticus* S2052, we found that the biosynthetic genes of one of the predicted type 3 polyketide synthase (T3PKS) BGCs was not differentially expressed when the strain was grown on chitin but that those from the remaining five predicted BGCs were either up- or downregulated (Table 1). In all cases but that of the andrimid BGC (see below), the change in gene expression was observed in the stationary phase. For one of the two predicted hybrid nonribosomal peptide type 1 PKSs (NRPS-T1PKS) and the siderophore and ectoine BGCs, some of the biosynthetic genes were upregulated, while others were downregulated. The biosynthetic genes of the second predicted NRPS-T1PKS clusters were downregulated, while those from the putative NRPS and the arylpolyene-NRPS BGC were upregulated. The latter encodes the machinery required for the biosynthesis of the acetyl coenzyme A (acetyl-CoA) carboxylase inhibitor andrimid, as suggested by the antiSMASH results and confirmed by BLAST search against the genome of *V. coralliilyticus* S2052 using the nucleotide sequence of the andrimid BGC from *Pantoea agglomerans* (16) as a query (not shown). While the andrimid biosynthetic genes were significantly upregulated only in the exponential phase (Table 1), the TW71_08085 gene, encoding a putative acetyl-CoA carboxylase carrying the single amino acid mutation M203L (not shown) required for andrimid resistance (17), was significantly upregulated in both exponential and stationary phase. The gene encoding a putative LysR family transcriptional regulator (TW71_08080) and located downstream of the andrimid biosynthetic genes was also slightly upregulated (fold change, 1.61).

**TABLE 1:** Range of the fold changes in expression of the biosynthetic genes contained in the BGCs predicted by antiSMASH in the genomes of *Vibrio coralliilyticus* S2052 and *Photobacterium galatheae* S2753

Strain	Cluster	Type of BGC	Range of FCs in expression of biosynthetic genes	Time point^a^
*V. coralliilyticus* S2052	1	T3PKS	X	X
	2	NRPS	2, 3.8	Stat
	3	Arylpolyene-NRPS (andrimid)	2.6, 6.8	Exp
	4	NRPS-T1PKS	−3, −18	Stat
	5	NRPS-T1PKS	−4.8, −7.6	Stat
	6	Ectoine	−2.2, 2.5	Stat
*P. galatheae* S2753	1	Other	X	X
	2	NRPS	X	X
	3	Other	X	X
	5	NRPS-T1PKS	2.2, 3	Exp
	6	NRPS	34.6	Stat
	7	Siderophore	X	X
	8	T1PKS	X	X
	9	Other	2.3, 3.4	Stat
	10	Ectoine	9	Stat
	11	NRPS (holomycin)	5, 10	Stat

a The time point at which the up- or downregulation was observed is indicated. Exp, time point in the exponential growth phase; Stat, time point in the stationary growth phase; X, not differentially expressed.

### The majority of BGCs of *P. galatheae* S2753 are upregulated on chitin compared to on glucose.

In *P. galatheae* S2753, the levels of expression of biosynthetic genes from five of the putative BGCs predicted by antiSMASH were not different when the strain was grown on chitin compared to on glucose. However, five BGCs (one NRPS-T1PKS, two NRPSs, one “other” BGC, and one ectoine BGC) were significantly upregulated on chitin, and no BGC was downregulated. As in the case of *V. coralliilyticus* S2052, one BGC was upregulated in the exponential phase, while four BGCs were upregulated only in the stationary phase (Table 1). By homology search, we identified the biosynthetic genes from one of the most upregulated putative NRPS BGCs as those required for the production of the antibiotic holomycin (not shown). The gene encoding the ArsR family transcriptional regulator (EA58_20500) that is located upstream of the holomycin biosynthetic genes was slightly downregulated (−1.45-fold change).

### Influence of chitin on the metabolite profiles of *V. coralliilyticus* S2052 and *P. galatheae* S2753.

The levels of andrimid and of the related compound moiramide detected in extracts from 24-h-old cultures of *V. coralliilyticus* S2052 grown on chitin were approximately 1.8-fold higher than those detected in extracts from cultures grown on glucose (Table 2), which is in agreement with the upregulation of the andrimid biosynthetic genes observed during exponential phase (Table 1). Solonamides and ngercheumicins were present at similar levels in extracts from cultures of *P. galatheae* S2753 grown on the two substrates. We identified two new members of the solonamide family (solonamides C and D) based on their accurate masses and retention times. These analogues differed from the known solonamides and ngercheumicins in their compositions and the orders of their constituent amino acids. The amino acid sequences of solonamides C and D could be tentatively assigned based on analysis of their tandem mass spectrometry (MS/MS) fragmentation pattern and by analogy with the observed fragments in the known solonamides (Fig. S2 to S4). Extracts from cultures grown in chitin contained approximately 0.5-fold-more holomycin than those from cultures grown in glucose (Table 2), which reflects the different expression levels of the related biosynthetic genes observed in the stationary phase. Several other possible secondary metabolites were found to occur in differing quantities in extracts of the cultures grown on the two substrates (Table S5), but we were not able to identify these compounds.

**TABLE 2:** Relative abundances of metabolites detected by LC-MS when strains were grown on chitin and glucose

Strain	Metabolite	Fold change^b^
*P. galatheae* S2753	Holomycin	0.5
	Solonamide	−0.2
	Ngercheumicin	−0.3
	Indigoidine^a^	ND
	Ectoine^a^	ND
*V. coralliilyticus* S2052	Andrimid	1.8
	Aerobactin^a^	ND

a The presence of these compounds was checked due to the antiSMASH prediction of the relative BGC in the genomes.

b ND, not detected.

## DISCUSSION

Studying the dynamics and evolution of the interactions between microorganisms and the surrounding environment is crucial for understanding their role in ecological systems. We studied the genetic potential of two members of the *Vibrionaceae* family for the utilization of chitin, the most abundant polymer in the marine environment (1), and analyzed at the transcriptional and metabolome levels their responses to the presence of this molecule. We analyzed the transcriptomes of the two strains grown on chitin and on glucose, a molecule that is also abundant in the marine environment (18). We performed this analysis during both the exponential and stationary growth phases, as we were interested in the temporal dynamics occurring during chitin colonization. We found that both strains possess the genetic information to produce a range of enzymes for chitin degradation and utilization and that their metabolite repertoires greatly vary when the strains are grown on chitin compared to on glucose, suggesting a role for the secondary metabolites during chitin colonization and utilization.

Most work on chitin utilization in *Vibrionaceae* has been done on a few *Vibrio* species, although the core gene set has been shown to be widespread in the whole family (3). Therefore, besides studying a *Vibrio* species (*V. coralliilyticus*), we studied a species from the genus* Photobacterium* (*P. galatheae*). The former is a coral pathogen (19), while the latter is a newly described species (20), and although it was isolated from a mussel (21), its preferred niche of colonization is unknown.

Both* V. coralliilyticus* S2052 and *P. galatheae* S2753 have the potential to produce a broad range of enzymes capable of binding and/or hydrolyzing chitin, chitin-derived molecules, and/or cellulose. Some of these enzymes contain the LPMO_10 domain, which is also present in GbpA (22), a colonization factor contributing to *V. cholerae* adhesion to chitinous surfaces (23). The putative cytoplasmic localization for all of the GH3 domain-containing proteins indicates that they may actually be involved in functions other than chitin degradation, as in the case for the GH3 hydrolase NagZ from *Salmonella enterica* serovar Typhimurium and *Bacillus subtilis*, which participate in peptidoglycan recycling (24). The organization of the *nag* operon in *V. coralliilyticus* S2052, in which, unlike in *Escherichia coli* (5, 6) and *P. galatheae*, the *nagB* gene is separated from the rest of the operon, resembles the organization of the same operon in *V. cholerae* (25, 26). Notably, the two different organizations of this operon in *V. coralliilyticus* S2052 and *P. galatheae* S2753 reflect those occurring in a number of other *Vibrio* and *Photobacterium* species, respectively (see Fig. S5 in the supplemental material). In contrast, the lack of homologs of the VC0611 (GlcNAc-1-phosphate mutase) and VC0612 [(GlcNAc)_2_ phosphorylase] genes in the (GlcNAc)_2_ operon of *P. galatheae* seems to be a peculiarity only of this strain and of the very closely related species *P. halotolerans* (Fig. S5). The differences in the organizations and contents of the operons indicate a certain degree of divergence during the evolution of the chitin utilization pathway in the different species. In *P. galatheae* S2753, given its ability to metabolize chitin despite its lack two genes from the (GlcNAc)_2_ operon, it is likely that other enzymes assist the reactions that in *V. cholerae* are catalyzed by the products of the VC0611 and VC0612 genes.

With respect to the dynamics of the chitin colonization and utilization program, the models currently available in the literature (see the introduction) cover the species used in this study, although for *P. galatheae* S2753, the poor annotation of the genome and the lack of complementary information in the literature and databases did not allow a thorough analysis. During exponential growth, *V. coralliilyticus* S2052 upregulated a number of genes encoding proteins putatively involved in chemotaxis (Table S4). The upregulation of a chitin-sensing chemotaxis machinery is undoubtedly an advantage during competition in a low-nutrient-containing environment or during the establishment of an association with a chitin-containing host. This is exemplified by the mutualistic relationship between *Vibrio fischeri* and the bobtail squid *Euprymna scolopes*, which is initiated by a gradient of chitin-derived oligosaccharides produced by the host (27). We also observed that a number of genes encoding transporters were upregulated. These were transporters involved in the uptake of chitin and chitin-derived oligosaccharides but also C_4_-dicarboxylate ABC transporters, and this upregulation was observed also in *P. galatheae* S2753 in the stationary phase. C_4_-dicarboxylate ABC transporters have different substrates, including sialic acids. Sialic acids are GlcNAc-derivative molecules used by mammals to glycosylate proteins on cell surfaces, where they act as determinants in bacterial adhesion events (28). Indeed, the ability to catabolize sialic acid is thought to be an important feature in *V. cholerae* during intestinal colonization (29). Some prokaryotes can produce sialic acids, and *V. coralliilyticus* BAA450 harbors the genes for their biosynthesis (30). However, the homologs of such genes in *V. coralliilyticus* S2052 were not differentially expressed in our experimental setup. Therefore, the upregulation of these transporters indicates that they may also use chitin-derived oligosaccharides as substrates or that *V. coralliilyticus* S2052 was deceived by the presence of GlcNAc and activated a response for the colonization of a potential host. The latter hypothesis is in agreement with the upregulation of genes encoding proteins containing one Hcp effector domain. In *V. cholerae*, Hcp proteins are involved in its virulence toward eukaryotic cells (31) and prokaryotic cells, conferring a competitive advantage to the producer (32). Furthermore, in *V. coralliilyticus* S2052, a set of genes (TW71_18705 to TW71_18720) annotated as “glycerol-3-phosphate dehydrogenase” were upregulated in both the exponential phase and the stationary phase. We believe that these genes were misannotated since their encoded products contain a RebB domain, necessary for the production of R-bodies. These are cytoplasmic inclusions occurring widely in *Proteobacteria* whose exact function is not known, but they have been suggested to play a role during host infection (33).

10.1128/mSystems.00141-16.4 TABLE S4  Fold changes (FC) of genes related to chemotaxis in *Vibrio coralliilyticus* S2052 and *Photobacterium galatheae* S2753 when the organisms were grown on chitin compared to glucose. Download TABLE S4, DOCX file, 0.02 MB. Copyright © 2017 Giubergia et al. 2017 Giubergia et al. This content is distributed under the terms of the Creative Commons Attribution 4.0 International license.

10.1128/mSystems.00141-16.5 TABLE S5  Fold change (chitin versus glucose) in the expression of unknown compounds determined by LC-MS. Download TABLE S5, DOCX file, 0.02 MB. Copyright © 2017 Giubergia et al. 2017 Giubergia et al. This content is distributed under the terms of the Creative Commons Attribution 4.0 International license.

Growth on chitin resulted in changes in the levels of expression of genes putatively related to the production of secondary metabolites. Expression changes of these genes (mostly upregulation) were seen in both strains and were most pronounced in stationary phase. This strongly indicates that the molecules produced by the biosynthetic machinery encoded by those genes may have ecological functions and may confer an advantage to the producers during chitin colonization, as it was suggested for andrimid (13). Secondary metabolites are thought to play several roles in nature, such as antagonism, inter- and/or intraspecies communication, and stress response. We speculate that the well-known antibacterial compounds holomycin (34) and andrimid (35) may serve to antagonize competitors and that the quorum-sensing-interfering molecules solonamides and ngercheumicins may be involved in intercellular communication since they can interfere with the quorum-sensing system of *Staphylococcus aureus* (36, 37); however, the elucidation of the exact function of the secondary metabolites identified in this work during chitin colonization requires further work. With regard to the transcriptional regulation of the biosynthesis of secondary metabolites, the upregulation of the andrimid biosynthetic genes and of the LysR family transcriptional regulator located just downstream of them supports the hypothesis that such a regulator may have an important regulatory role in andrimid biosynthesis (38). Similarly, the downregulation of the ArsR family transcriptional regulator gene located upstream of the holomycin biosynthetic genes may indicate a role of the encoded regulator as a repressor of holomycin production. For most of the remaining differentially expressed biosynthetic genes, however, we do not know the metabolite produced by the proteins that they encode, even after comparing transcriptomics and metabolomics data.

In conclusion, the results of this study show that growth on chitin triggers a comprehensive response at the transcriptional and biosynthetic levels in vibrios, providing insights into the dynamics of colonization of chitinous surfaces in nature. We showed that the upregulation of genes related to the production of secondary metabolites is reflected in the metabolite profile, suggesting a role for these molecules during chitin colonization. Additional work, possibly in experimental setups with live chitin-containing zooplankton, may help to identify the function of this molecule in natural settings.

## MATERIALS AND METHODS

### Genome analyses.

A list of genes possibly involved in chitin utilization was compiled based on the analysis of the genomes of *Vibrio coralliilyticus* S2052 and *Photobacterium galatheae* S2753 (RefSeq accession numbers JXXR01 and JMIB01, respectively). The choice of the genes to be included in the list was made based on (i) the NCBI gene annotation list associated with each genome, (ii) homology searches using genes known to be related to chitin metabolism as queries, and (iii) the presence of Pfam and/or InterPro domains related to chitin metabolism in the amino acid sequences encoded by the genes. Pfam and InterPro domains were obtained by running through Blast2GO (39) the amino acid sequences encoded by all predicted genes in each genome. Amino acid sequences of the identified proteins were also submitted to the bacterial protein subcellular localization prediction tool (PSORTb 3.0) (40) to identify signal peptides linked to specific cellular compartments. Furthermore, genomes were submitted to antiSMASH 3.0 (15) for the prediction of a putative biosynthetic gene cluster involved in the production of secondary metabolites.

### Bacterial strains and medium composition.

*Photobacterium galatheae* S2753 and *Vibrio coralliilyticus* S2052 were isolated during the Galathea 3 global research expedition (21). The compositions of the media used in this work were 2% Sigma sea salts solution (catalog no. S9883; Sigma) with 0.3% Casamino Acids (catalog no. BD223050) supplemented with either 40 mM morpholinepropanesulfonic acid (MOPS; pH 7.5) and 0.2% glucose (SSBG) or 0.2% colloidal chitin (SSBC). Colloidal chitin was prepared as described previously (41).

### Growth conditions.

Unless stated otherwise, all cultures in liquid medium were grown aerated (200 rpm) at 25°C in four biological replicates. Single colonies of *P. galatheae* S2753 or *V. coralliilyticus* S2052 were grown in 10 ml of half-strength yeast extract-tryptone-sea salts medium (1/2 YTSS medium) (42) for 24 h. One hundred microliters of each culture was then used to inoculate 10 ml of fresh 1/2 YTSS medium. After 24 h, each culture was used to inoculate 50 ml of SSBG or SSBC in 250-ml Erlenmeyer flasks at approximately 10^3^ CFU/ml. When cultures reached late exponential and early stationary phase (see Fig. S1 in the supplemental material), a subsample was taken, mixed with 0.2 volumes of ice-cold stop solution (95% [vol/vol] ethanol, 5% [vol/vol] phenol), incubated on ice for 5 min, and pelleted by centrifugation. Supernatants were removed, and cell pellets were stored at −80°C until RNA extraction.

10.1128/mSystems.00141-16.6 FIG S1  Growth curves of *Vibrio coralliilyticus* S2052 (top) and *Photobacterium galatheae* S2753 (bottom) in glucose (SSBG, triangles) and chitin (SSBC, squares). The beige and light-green time points indicate the harvest points in the late exponential and in the stationary phase, respectively. Download FIG S1, DOCX file, 0.1 MB. Copyright © 2017 Giubergia et al. 2017 Giubergia et al. This content is distributed under the terms of the Creative Commons Attribution 4.0 International license.

10.1128/mSystems.00141-16.7 FIG S2  HRMS-MS spectra of solonamide C. Download FIG S2, DOCX file, 0.1 MB. Copyright © 2017 Giubergia et al. 2017 Giubergia et al. This content is distributed under the terms of the Creative Commons Attribution 4.0 International license.

10.1128/mSystems.00141-16.8 FIG S3  HRMS-MS spectra of solonamide D. Download FIG S3, DOCX file, 0.1 MB. Copyright © 2017 Giubergia et al. 2017 Giubergia et al. This content is distributed under the terms of the Creative Commons Attribution 4.0 International license.

10.1128/mSystems.00141-16.9 FIG S4  Proposed major MS/MS fragments of solonamide C (A) and D (B). Download FIG S4, DOCX file, 0.2 MB. Copyright © 2017 Giubergia et al. 2017 Giubergia et al. This content is distributed under the terms of the Creative Commons Attribution 4.0 International license.

10.1128/mSystems.00141-16.10 FIG S5  (A) Distribution of the *nag* operon in *Vibrio* species; (B) distribution of the *nag* operon in *Photobacterium* species; (C) distribution of GlcNAc_2_ in *Photobacterium* species. Homology searches were done by using MultiGeneBlast (44). Download FIG S5, DOCX file, 0.4 MB. Copyright © 2017 Giubergia et al. 2017 Giubergia et al. This content is distributed under the terms of the Creative Commons Attribution 4.0 International license.

### RNA isolation and sequencing

RNA was extracted using the RNeasy kit (catalog no. 74104; Qiagen) by following the manufacturer’s instructions. DNA was removed on-column with the RNase-free DNase set (catalog no. 79254; Qiagen). The integrity and quality of total RNA were assessed with a NanoDrop Spectrometer (Saveen Werner, Sweden) and an Agilent 2100 Bioanalyzer (Agilent Technologies). For each strain under each condition, the three best total RNA samples were sent to the Beijing Genome Institute (BGI; Hong Kong, China), where rRNA was removed using the Ribo-Zero rRNA removal kit (Illumina). Libraries were then constructed with the TruSeq RNA library preparation kit (Illumina) and their 100-bp paired ends were sequenced on a HiSeq 2000.

### Data analysis.

RNA-seq data were analyzed using CLC Genomics Workbench version 8 (CLC Bio, Aarhus, Denmark). Quality control of the reads was done based on the %GC, Phred score, nucleotide contribution, and enriched 5-mer sequences. Reads were trimmed by removing the first 15 nucleotides from the 5′ end when nucleotide contribution was not normally distributed. Subsequently, reads were mapped to the reference genomes and expression values were calculated as reads per kilobase per million mapped reads (RPKM). Gene expression profiles of biological replicates were merged, and for each strain at each time point, the profiles deriving from the two media were compared. The quality of the transcriptomic data was evaluated using hierarchal clustering and principal-component analysis. Data sets that did not pass the quality control were discarded; however, no less than two biological replicates per strain per condition per data point were used. Statistically significant gene expression differences were assessed through Baggerly et al.’s test (43) using a *P* value of <0.05 and a false discovery rate (*q* value) of <0.05. For the analysis of the global transcription profiles, only genes with an absolute fold change of >5 were considered. For other analyses (chitin utilization-related genes and secondary metabolism biosynthetic genes), no fold change limit was set.

### Extraction of liquid cultures for chemical analysis.

Subsamples (2 ml) of the 24-h-old cultures (harvest point in the stationary phase) were collected to be used for secondary metabolite analysis. Cultures were extracted sequentially with an equal volume of high-performance liquid chromatography (HPLC)-grade ethyl acetate (EtOAc) (neutral extract), EtOAc containing 1% formic acid (acidic extract), and then EtOAc containing 2% ammonia (basic extract). The organic phases were transferred to fresh vials and evaporated to dryness under a stream of nitrogen. Extracts were dissolved in 250 μl methanol (MeOH) and stored at −20°C. The neutral, acidic, and basic extracts were kept separate and analyzed separately. For each species, three biological replicates and two technical replicates were analyzed.

### UHPLC-HRMS.

Ultrahigh-performance liquid chromatography–high-resolution mass spectrometry (UHPLC-HRMS) was performed on an Agilent Infinity 1290 UHPLC system (Agilent Technologies, Santa Clara, CA) equipped with a diode array detector. Separation was obtained on an Agilent Poroshell 120 phenyl-hexyl column (2.1 by 250 mm; particle size, 2.7 μm) with a linear gradient consisting of water and acetonitrile, both buffered with 20 mM formic acid, starting at 10% acetonitrile and increasing to 100% in 15 min, at which point the concentration was held for 2 min, returned to 10% acetonitrile in 0.1 min, and left for 3 min (0.35 ml/min, 60°C). An injection volume of 1 μl was used. MS detection was performed in both the positive and negative detection modes on an Agilent 6540 quadrupole time of flight (QTOF) MS equipped with an Agilent dual-jet-stream electrospray ion source with a drying gas temperature of 250°C, a gas flow of 8 liters/min, a sheath gas temperature of 300°C, and a flow rate of 12 liters/min. Capillary voltage was set to 4,000 V and nozzle voltage to 500 V. Mass spectra were recorded at 10, 20, and 40 eV as centroid data for *m/z* 85 to 1,700 in MS mode and for *m/z* 30 to 1,700 in MS/MS mode, with an acquisition rate of 10 spectra/s. Lock mass solution in 70:30 methanol-water was infused into the second sprayer using an extra LC pump at a flow rate of 15 μl/min and a 1:100 splitter. The solution contained 1 μM tributylamine (Sigma-Aldrich) and 10 μM hexakis(2,2,3,3-tetrafluoropropoxy)phosphazene (Apollo Scientific Ltd., Cheshire, United Kingdom) as lock masses. The [M + H]^+^ ions (*m/z* 186.2216 and 922.0098, respectively) of both compounds were used.

### Dereplication.

The extracts were dereplicated by searching by formulas for all compounds known to be produced by *Vibrio* and *Photobacterium* species found in Antibase 2012 (https://www.chemits.com/en/software/chemical-databases/antibase.html), MarinLit 2012 (http://pubs.rsc.org/marinlit/), and the Dictionary of Natural Products (http://dnp.chemnetbase.com/dictionary-search.do;jsessionid=C5FC42DCF729956FDF9BF3B79BF47CDA?method=view&id=11964904&si=). The chromatograms were then examined for peaks of intensity which correlated with the change in expression levels revealed by the transcriptomic data. Unknown compounds of potential interest were analyzed by examination of the MS/MS data to assist with identification of the compound class and derive structural information.

### Compound quantification.

Andrimid was quantified using the sum of the [M + Na]^+^ and [2M + Na]^+^ ions (*m/z* 502.2312 and 981.4733, respectively). Holomycin was quantified using the sum of the [M + H]^+^ and [M + Na]^+^ ions (*m/z* 214.9943 and 236.9763). The solonamides were quantified as a sum of their respective [M + H]^+^ and [M + Na]^+^ ions (solonamide A, *m/z* 559.3490 and 581.3310; solonamide B, *m/z* 587.3803 and 609.3623; solonamide C. *m/z* 573.3647 and 595.3466; solonamide D, *m/z* 573.3647 and 595.3466). The ngercheumicins also were quantified as the sum of their respective [M + H]^+^ and [M + Na]^+^ ions (ngercheumicin A, *m/z* 825.5332 and 847.5151; ngercheumicin B, *m/z* 827.5488 and 849.5308; ngercheumicin F, *m/z* 854.5723 and 876.5543; ngercheumicin G, *m/z* 856.5880 and 878.5699; ngercheumicin H, *m/z* 881.5958 and 903.5777; ngercheumicin I, *m/z* 883.6114 and 905.5934). The change in metabolite abundance was assessed for statistical significance using Student’s *t* test with an alpha level of 0.05.

### Accession number(s).

Sequencing data have been deposited in the Gene Expression Omnibus (GEO) database under GenBank accession number GSE80783.
