# Supplementary material for: Growth on Chitin Impacts the Transcriptome and Metabolite Profiles of Antibiotic-Producing Vibrio coralliilyticus S2052 and Photobacterium galatheae S2753
Source: mSystems. 2017 Jan 3;2(1):e00141-16. doi: 10.1128/mSystems.00141-16 (PMC5209532; doi:10.1128/mSystems.00141-16)
Supplement: TABLE S1 [file sys001172077st1.docx]

**Table SI1** **Gene lis**t. List of genes identified in the genomes of *Vibrio coralliilyticus* S2052 and *Photobacterium galatheae* S2753 and encoding for proteins containing Pfam domains related to the binding or the hydrolysis of chitin and cellulose. The predicted cellular localization is indicated as well. DUF: domain of unknown function.
