# Supplementary material for: Growth on Chitin Impacts the Transcriptome and Metabolite Profiles of Antibiotic-Producing Vibrio coralliilyticus S2052 and Photobacterium galatheae S2753
Source: mSystems. 2017 Jan 3;2(1):e00141-16. doi: 10.1128/mSystems.00141-16 (PMC5209532; doi:10.1128/mSystems.00141-16)
Supplement: TABLE S2 [file sys001172077st2.docx]

**Table SI2** **antiSMASH results**. antiSMASH prediction of the genetic potential of *Vibrio coralliilyticus* S2052 and *Photobacterium galatheae* S2753 for the biosynthesis of secondary metabolites. The table lists the type of putative biosynthetic gene cluster (BGC), the locus tags of the genes predicted to be part of them and, when known, the most similar known biosynthetic gene cluster. T3PKS: type 3 polyketide synthase; NRPS: non ribosomal peptide synthase; T1PKS: type 1 polyketide synthase.
