# Supplementary material for: Growth on Chitin Impacts the Transcriptome and Metabolite Profiles of Antibiotic-Producing Vibrio coralliilyticus S2052 and Photobacterium galatheae S2753
Source: mSystems. 2017 Jan 3;2(1):e00141-16. doi: 10.1128/mSystems.00141-16 (PMC5209532; doi:10.1128/mSystems.00141-16)
Supplement: TABLE S3 [file sys001172077st3.docx]

**Table SI3 Most upregulated genes.** The ten most up and down regulated genes for each strain in each condition at the two sampling points (late exponential and stationary phase). Values refer to chitin compared to glucose. Up: upregulated; down: downregulated; FC: fold change

| Sample | Gene | FC | PGAP annotation |
| --- | --- | --- | --- |
| *V. coralliilyticus* S2052 exponential phase up | TW71_04295 | 196 | aldehyde dehydrogenase |
|  | TW71_05770 | 89 | C4-dicarboxylate ABC transporter |
|  | TW71_07295 | 81 | acetyl-CoA synthetase |
|  | TW71_07940 | 79 | hypothetical protein |
|  | TW71_19770 | 70 | hypothetical protein |
|  | TW71_00320 | 68 | hypothetical protein |
|  | TW71_07870 | 63 | hypothetical protein |
|  | TW71_00325 | 57 | dehydrogenase |
|  | TW71_07945 | 51 | hypothetical protein |
|  | TW71_18840 | 50 | hypothetical protein |
| *V. coralliilyticus* S2052 stationary phase up | TW71_19275 | 159 | peptide ABC transporter substrate-binding protein |
|  | TW71_19270 | 140 | peptide ABC transporter permease |
|  | TW71_18580 | 140 | Trp operon leader peptide |
|  | TW71_14655 | 103 | amino acid deaminase |
|  | TW71_23380 | 85 | chitinase |
|  | TW71_16830 | 83 | 5-methyltetrahydropteroyltriglutamate-- homocysteine methyltransferase |
|  | TW71_19265 | 75 | peptide ABC transporter substrate-binding protein |
|  | TW71_15905 | 68 | hypothetical protein |
|  | TW71_19260 | 63 | sugar ABC transporter ATP-binding protein |
|  | TW71_19250 | 57 | chitobiase |
| *P. galatheae* S2753 exponential phase up | EA58_07425 | 39 | dethiobiotin synthetase |
|  | EA58_12415 | 21 | formimidoylglutamase |
|  | EA58_12420 | 17 | urocanate hydratase |
|  | EA58_12425 | 17 | histidine ammonia-lyase |
|  | EA58_20235 | 12 | hypothetical protein |
|  | EA58_07075 | 12 | hypothetical protein |
|  | EA58_10775 | 12 | glyceraldehyde-3-phosphate dehydrogenase |
|  | EA58_18255 | 12 | thioesterase |
|  | EA58_12410 | 11 | imidazolonepropionase |
|  | EA58_16300 | 9 | hypothetical protein |
| *P. galatheae* S2753 stationary phase up | EA58_20785 | 799 | hypothetical protein |
|  | EA58_20780 | 648 | benzoylformate decarboxylase |
|  | EA58_06000 | 239 | glycosyltransferase |
|  | EA58_06005 | 150 | hypothetical protein |
|  | EA58_21470 | 137 | lactonizing lipase |
|  | EA58_15365 | 102 | hemolysin |
|  | EA58_16750 | 94 | hypothetical protein |
|  | EA58_07035 | 90 | chitodextrinase |
|  | EA58_12040 | 73 | phosphonate ABC transporter substrate-binding protein |
|  | EA58_19900 | 61 | chitinase |
| *V. coralliilyticus* S2052 exponential phase down | TW71_11480 | -50 | maltoporin |
|  | TW71_00570 | -34 | hypothetical protein |
|  | TW71_14985 | -33 | maltodextrin phosphorylase |
|  | TW71_14690 | -28 | cytochrome O ubiquinol oxidase |
|  | TW71_14695 | -24 | cytochrome o ubiquinol oxidase subunit I |
|  | TW71_04930 | -23 | oxalate:formate antiporter |
|  | TW71_14990 | -22 | 4-alpha-glucanotransferase |
|  | TW71_14700 | -19 | cytochrome o ubiquinol oxidase subunit III |
|  | TW71_14705 | -17 | cytochrome O ubiquinol oxidase |
|  | TW71_14095 | -16 | glyoxalase |
| *V. coralliilyticus* S2052 stationary phase down | TW71_00500 | -2273 | secretion protein |
|  | TW71_00505 | -2235 | type III secretion protein |
|  | TW71_00510 | -2115 | secretion protein EspA |
|  | TW71_00495 | -1833 | pathogenicity island effector protein |
|  | TW71_00490 | -1197 | type III secretion protein |
|  | TW71_00515 | -990 | secretion protein EspA |
|  | TW71_00485 | -693 | hypothetical protein |
|  | TW71_00345 | -551 | hypothetical protein |
|  | TW71_00355 | -275 | hypothetical protein |
|  | TW71_00430 | -249 | type III secretion system needle protein SsaG |
| *P. galatheae* S2753 exponential phase down | EA58_17070 | -17 | glutamate synthase |
|  | EA58_02025 | -15 | hypothetical protein |
|  | gltD | -15 |  |
|  | EA58_16650 | -12 | cytochrome D ubiquinol oxidase subunit I |
|  | EA58_07025 | -11 | hypothetical protein |
|  | EA58_19405 | -10 | acetate kinase |
|  | EA58_07020 | -9 | cytochrome o ubiquinol oxidase subunit I |
|  | EA58_07015 | -8 | cytochrome o ubiquinol oxidase subunit III |
|  | EA58_17025 | -8 | lactoylglutathione lyase |
|  | EA58_07010 | -7 | cytochrome O ubiquinol oxidase |
| *P. galatheae* S2753 stationary phase down | EA58_06945 | -47 | flagellar basal body rod protein FlgC |
|  | EA58_07025 | -47 | hypothetical protein |
|  | EA58_00065 | -37 | hypothetical protein |
|  | EA58_12415 | -30 | formimidoylglutamase |
|  | EA58_07020 | -26 | cytochrome o ubiquinol oxidase subunit I |
|  | flgB_2 | -24 |  |
|  | EA58_12410 | -23 | imidazolonepropionase |
|  | EA58_20875 | -23 | hypothetical protein |
|  | EA58_01880 | -21 | acetate kinase |
|  | EA58_11270 | -21 | hypothetical protein |
