# Supplementary material for: Growth on Chitin Impacts the Transcriptome and Metabolite Profiles of Antibiotic-Producing Vibrio coralliilyticus S2052 and Photobacterium galatheae S2753
Source: mSystems. 2017 Jan 3;2(1):e00141-16. doi: 10.1128/mSystems.00141-16 (PMC5209532; doi:10.1128/mSystems.00141-16)
Supplement: TABLE S4 [file sys001172077st4.docx]

**Table SI4 Chemotaxis genes.** Fold changes (FC) of genes related to chemotaxis in *Vibrio coralliilyticus* S2052 and *Photobacterium galatheae* S2753 when the organisms are grown on chitin as compared to glucose.

|  | Gene | FC | PGAP annotation |
| --- | --- | --- | --- |
| *V. coralliilyticus* S2052 exponential phase | TW71_01215 | 2,4 | chemotaxis protein CheY |
|  | TW71_01445 | -2,1 | chemotaxis protein |
|  | TW71_05740 | 3,7 | chemotaxis protein CheY |
|  | TW71_06500 | -5,9 | chemotaxis protein |
|  | TW71_07880 | 2,5 | chemotaxis protein CheY |
|  | TW71_09420 | 2,3 | chemotaxis protein CheY |
|  | TW71_11490 | 21,1 | chemotaxis protein |
|  | TW71_11670 | 3,5 | chemotaxis protein |
|  | TW71_13190 | 2,2 | chemotaxis protein |
|  | TW71_14260 | 2,2 | chemotaxis protein CheY |
|  | TW71_14270 | 2,6 | chemotaxis protein |
|  | TW71_14275 | 4,2 | chemotaxis protein |
|  | TW71_14290 | 2,9 | chemotaxis protein |
|  | TW71_14295 | 3,9 | chemotaxis protein CheW |
|  | TW71_14305 | 5,9 | chemotaxis protein CheA |
|  | TW71_14320 | 4,2 | chemotaxis protein |
|  | TW71_14940 | 9,5 | chemotaxis protein |
|  | TW71_15635 | -4,8 | chemotaxis protein |
|  | TW71_16580 | 2,7 | chemotaxis protein CheX |
|  | TW71_18425 | 2,2 | chemotaxis protein |
|  | TW71_18985 | 7,8 | chemotaxis protein CheY |
|  | TW71_19075 | 8,1 | chemotaxis protein |
|  | TW71_19255 | 27,2 | chemotaxis protein |
|  | TW71_24705 | 4,5 | chemotaxis protein |
| *V. coralliilyticus* S2052 stationary phase | TW71_07720 | 2,2 | chemotaxis protein |
|  | TW71_11490 | -2,1 | chemotaxis protein |
|  | TW71_15165 | 2,0 | chemotaxis protein |
|  | TW71_19255 | 50,3 | chemotaxis protein |
|  | TW71_24705 | -2,1 | chemotaxis protein |
| *P. galatheae* S2753 exponential phase | EA58_05570 | 2,2 | chemotaxis protein CheY |
|  | EA58_15700 | -2,9 | chemotaxis protein |
| *P. galatheae* S2753 stationary phase | EA58_01610 | -5,1 | chemotaxis protein CheY |
|  | EA58_02020 | 4,1 | chemotaxis protein |
|  | EA58_02250 | -2,2 | chemotaxis protein CheW |
|  | EA58_02255 | -2,2 | chemotaxis protein CheR |
|  | EA58_02470 | 2,4 | chemotaxis protein CheA |
|  | EA58_05760 | 2,3 | chemotaxis protein |
|  | EA58_07330 | 2,9 | chemotaxis protein |
|  | EA58_07600 | -4,2 | chemotaxis protein |
|  | EA58_10710 | 4,6 | chemotaxis protein |
|  | EA58_16910 | 7,9 | chemotaxis protein |
|  | EA58_18325 | 3,7 | chemotaxis protein |
