# Supplementary material for: Growth on Chitin Impacts the Transcriptome and Metabolite Profiles of Antibiotic-Producing Vibrio coralliilyticus S2052 and Photobacterium galatheae S2753
Source: mSystems. 2017 Jan 3;2(1):e00141-16. doi: 10.1128/mSystems.00141-16 (PMC5209532; doi:10.1128/mSystems.00141-16)
Supplement: TABLE S5 [file sys001172077st5.docx]

**Table SI5 Compounds fold changes.** Fold change **(**chitin versus glucose) of unknown compounds determined by LC-MS.

|  | **Detected *m/z*** | **Proposed Formula (neutral)** | **Fold Change** |
| --- | --- | --- | --- |
| *P. galatheae* S2753 | 219.1225 | C_10_H_18_O_5_ | 16.6 |
|  | 233.1018 | C_10_H_16_O_6_ | 9.2 |
|  | 599.392 | C_32_H_50_N_6_O_5_ | -0.7 |
|  | 305.1591 | C_14_H_24_O_7_ | Not detected on glucose |
|  | 316.212 | C_16_H_29_NO_5_ | -0.7 |
|  | 619.3809 | C_30_H_54_N_2_O_11_ | Not detected on glucose |
| *V. coralliilyticus* S2052 | 294.12 | C_11_H_19_NO_8_ | 7.6 |
|  | 365.3166 | C_22_H_40_N_2_O_2_ | 7.8 |
