# Supplementary material for: Growth on Chitin Impacts the Transcriptome and Metabolite Profiles of Antibiotic-Producing Vibrio coralliilyticus S2052 and Photobacterium galatheae S2753
Source: mSystems. 2017 Jan 3;2(1):e00141-16. doi: 10.1128/mSystems.00141-16 (PMC5209532; doi:10.1128/mSystems.00141-16)
Supplement: FIG S1 [file sys001172077sf6.docx]

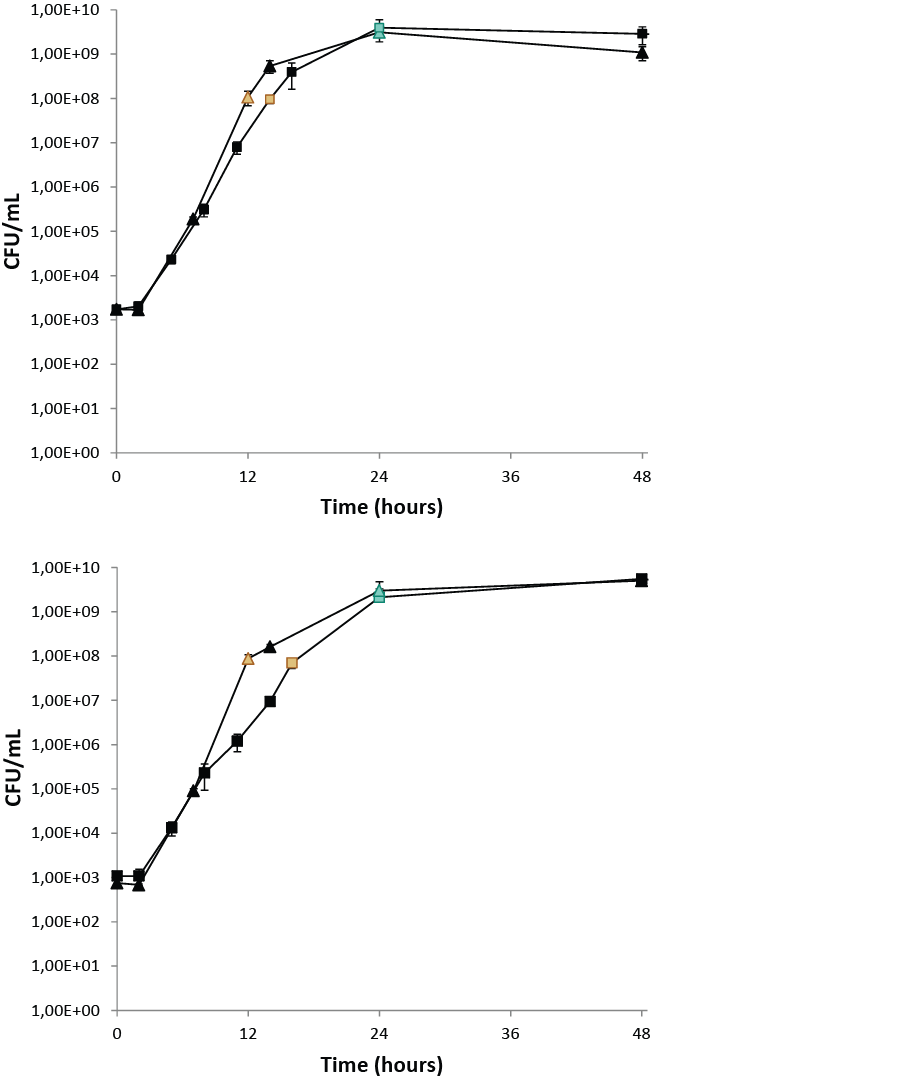


**Figure SI1** **Growth curves**. Growth curves of *Vibrio coralliilyticus* S2052 (top) and *Photobacterium galatheae* S2753 (bottom) in glucose (SSBG triangles) and chitin (SSBC squares). The beige and light green time points indicate the harvest points in the late exponential and in the stationary phase, respectively.
