# Supplementary material for: Growth on Chitin Impacts the Transcriptome and Metabolite Profiles of Antibiotic-Producing Vibrio coralliilyticus S2052 and Photobacterium galatheae S2753
Source: mSystems. 2017 Jan 3;2(1):e00141-16. doi: 10.1128/mSystems.00141-16 (PMC5209532; doi:10.1128/mSystems.00141-16)
Supplement: FIG S2 [file sys001172077sf7.docx]

| Name | Observed mass | Predicted formula | Predicted Mass | Error (ppm) |
| --- | --- | --- | --- | --- |
| Solonamide C |  | C_31_H_48_N_4_O_6_ | 572.3574 |  |
| M+Na | 595.3473 | C_31_H_48_N_4_O_6_Na^+^ | 595.3466 | 1.18 |
| M+H | 573.366 | C_31_H_49_N_4_O_6_^+^ | 573.3647 | 2.27 |
|  | 545.3697 | C_30_H_49_N_4_O_5_^+^ | 545.3697 | 0 |
|  | 460.2805 | C_25_H_38_N_3_O_5_^+^ | 460.2806 | -0.22 |
|  | 432.2854 | C_24_H_38_N_3_O_4_^+^ | 432.2857 | -0.69 |
|  | 403.2597 | C_23_H_35_N_2_O_4_^+^ | 403.2591 | 1.49 |
|  | 385.2462 | C_18_H_33_N_4_O_5_^+^ | 385.2445 | 4.41 |
|  | 347.1968 | C_19_H_27_N_2_O_4_^+^ | 347.1965 | 0.86 |
|  | 290.1752 | C_17_H_24_NO_3_^+^ | 290.1751 | 0.34 |
|  | 262.1796 | C_16_H_24_NO_2_^+^ | 262.1802 | -2.29 |
|  | 120.0801 | C_8_H_10_N^+^ | 120.0808 | -5.83 |
|  | 86.0963 | C_5_H_12_N^+^ | 86.0964 | -1.16 |

**Figure SI2 HRMS/MS spectra.** HRMS/MS spectra of solonamide C.
