# Supplementary material for: Growth on Chitin Impacts the Transcriptome and Metabolite Profiles of Antibiotic-Producing Vibrio coralliilyticus S2052 and Photobacterium galatheae S2753
Source: mSystems. 2017 Jan 3;2(1):e00141-16. doi: 10.1128/mSystems.00141-16 (PMC5209532; doi:10.1128/mSystems.00141-16)
Supplement: FIG S3 [file sys001172077sf8.docx]

| Name | Observed Mass | Predicted Formula | Predicted Mass | Error (ppm) |
| --- | --- | --- | --- | --- |
| Solonamide D |  | C_33_H_52_N_4_O_6_ | 600.3887 |  |
| M+Na | 623.3779 | C_33_H_52_N_4_O_6_Na^+^ | 623.3779 | 0 |
| M+H | 601.396 | C_33_H_53_N_4_O_6_^+^ | 601.396 | 0 |
|  | 573.3993 | C_32_H_53_N_4_O_5_^+^ | 573.401 | -2.96 |
|  | 488.3115 | C_27_H_42_N_3_O_5_^+^ | 488.3119 | -0.82 |
|  | 460.3161 | C_26_H_42_N_3_O_4_^+^ | 460.317 | -1.96 |
|  | 417.2743 | C_24_H_37_N_2_O_4_^+^ | 417.2748 | -1.20 |
|  | 399.2656 | C_24_H_35_N_2_O_3_^+^ | 399.2642 | 3.51 |
|  | 304.191 | C_18_H_26_NO_3_^+^ | 304.1907 | 0.99 |
|  | 286.1803 | C_18_H_24_NO_2_^+^ | 286.1802 | 0.35 |
|  | 185.128 | C_9_H_17_N_2_O_2_^+^ | 185.1285 | -2.70 |
|  | 120.0803 | C_8_H_10_N^+^ | 120.0808 | -4.16 |
|  | 86.0962 | C_5_H_12_N^+^ | 86.0964 | -2.32 |

**Figure SI3 HRMS/MS spectra.** HRMS/MS spectra of solonamide D.
