# Supplementary material for: Growth on Chitin Impacts the Transcriptome and Metabolite Profiles of Antibiotic-Producing Vibrio coralliilyticus S2052 and Photobacterium galatheae S2753
Source: mSystems. 2017 Jan 3;2(1):e00141-16. doi: 10.1128/mSystems.00141-16 (PMC5209532; doi:10.1128/mSystems.00141-16)
Supplement: FIG S4 [file sys001172077sf9.docx]

A)

B)

**Figure SI4** **MS/MS fragments**. Proposed major MS/MS fragments of solonamide C (A) and D (B).
