# Supplementary material for: Growth on Chitin Impacts the Transcriptome and Metabolite Profiles of Antibiotic-Producing Vibrio coralliilyticus S2052 and Photobacterium galatheae S2753
Source: mSystems. 2017 Jan 3;2(1):e00141-16. doi: 10.1128/mSystems.00141-16 (PMC5209532; doi:10.1128/mSystems.00141-16)
Supplement: FIG S5 [file sys001172077sf10.docx]

**
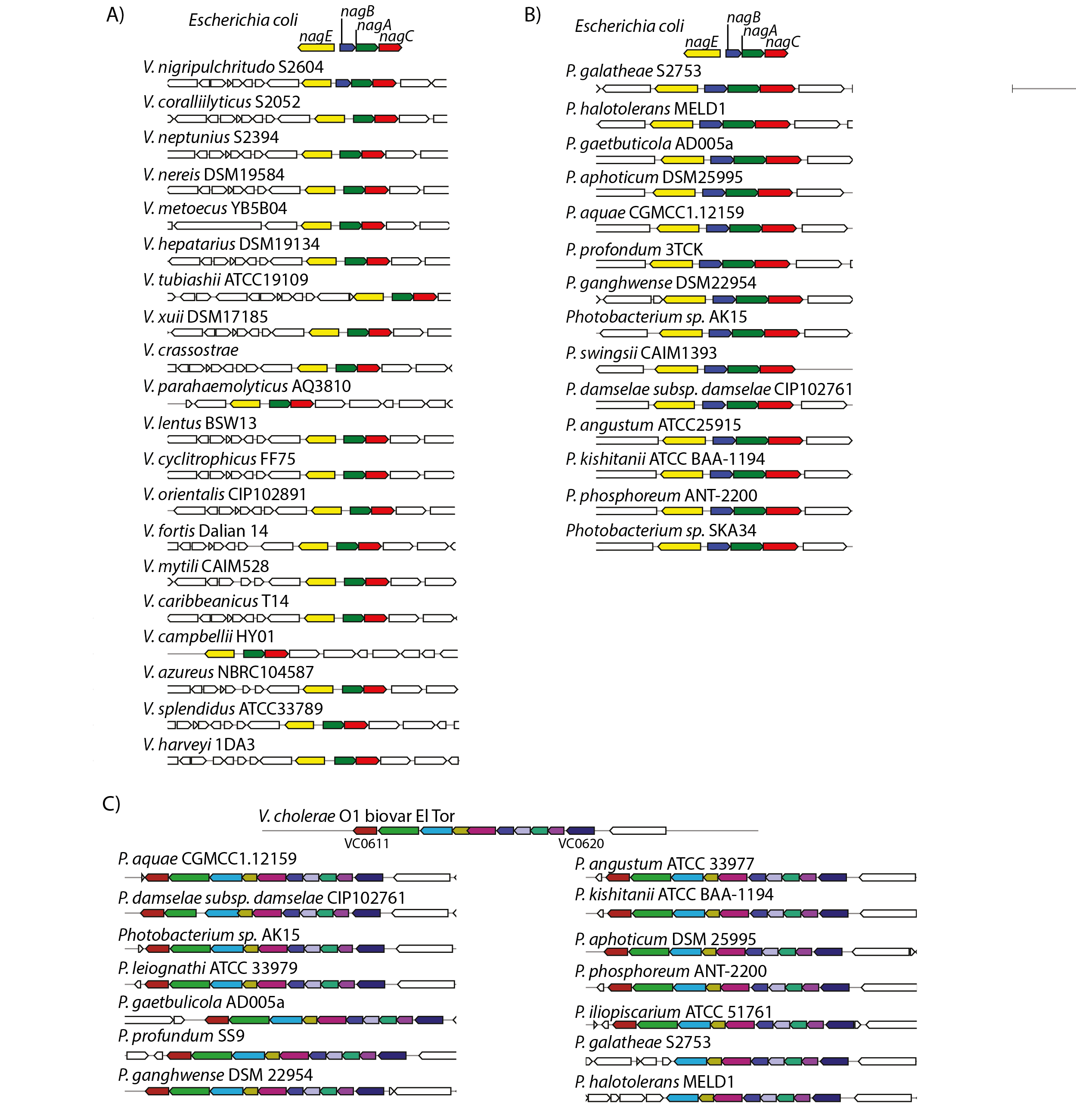
**

**Figure SI5** **Operons distribution.** A) Distribution of the *nag* operon in *Vibrio* species. B) Distribution of the *nag* operon in *Photobacterium* species. C) Distribution of the GlcNAc_2_ in *Photobacterium* species. Homology searches was done by MultiGeneBlast (Medema *et al.*, 2013).

**References**

Medema, M.H., Takano, E., and Breitling, R. (2013) Detecting Sequence Homology at the Gene Cluster Level with MultiGeneBlast. *Mol. Biol. Evol.* **30**: 1218–1223.
